# Towards Measuring Brain Function on Groups of People in the Real World

**DOI:** 10.1371/journal.pone.0044676

**Published:** 2012-09-05

**Authors:** Alan Gevins, Cynthia S. Chan, Lita Sam-Vargas

**Affiliations:** San Francisco Brain Research Institute & SAM Technology, San Francisco, California, United States of America; University College London, United Kingdom

## Abstract

In three studies, EEGs from three groups of participants were recorded during progressively more real world situations after drinking alcoholic beverages that brought breath alcohol contents near the limit for driving in California 30 minutes after drinking. A simple equation that measured neurophysiological effects of alcohol in the first group of 15 participants performing repetitive cognitive tasks was applied to a second group of 15 operating an automobile driving simulator, and to a third group of 10 ambulatory people recorded simultaneously during a cocktail party. The equation derived from the first group quantified alcohol’s effect by combining measures of higher frequency (beta) and lower frequency (theta) power into a single score. It produced an Area Under the Receiver Operator Characteristic Curve of .73 (p<.05; 67% sensitivity in recognizing alcohol and 87% specificity in recognizing placebo). Applying the same equation to the second group operating the driving simulator, AUC was .95, (p<.0001; 93% sensitivity and 73% specificity), while for the cocktail party group AUC was .87 (p<.01; 80% sensitivity and 80% specificity). EEG scores were significantly related to breath alcohol content in all studies. Some individuals differed markedly from the overall response evident in their respective groups. The feasibility of measuring the neurophysiological effect of a psychoactive substance from an entire group of ambulatory people at a cocktail party suggests that future studies may be able to fruitfully apply brain function measures derived under rigorously controlled laboratory conditions to assess drug effects on groups of people interacting in real world situations.

## Introduction

Characterizing the neurophysiological effects of drugs with EEGs is well established [1]-[2]. The question naturally arises how drug effects measured in the laboratory extend to real world situations. Such effects have rarely been measured outside the lab, for instance in vehicles being driven by fatigued and/or medicated drivers [3]-[4]. Further, since so much of human experience involves social interactions, an additional frontier is to extend brain function measurements to groups of people simultaneously. Progress has been made in this regard with EEG and fMRI measures [5]-[8], but neurophysiological effects of drugs have not been measured from more than one person at a time. Again, the frontier is to extend from contrived laboratory conditions to studies in real world situations.

Here we report initial progress in this regard in three studies in which an EEG measure of the effect of a psychoactive substance during highly controlled repetitive cognitive testing in the lab was applied first to the more complex activity of operating an automobile driving simulator and then to a real world situation in which EEGs were recorded concurrently from 10 ambulatory people during a cocktail party. To establish feasibility, a strong effect was studied across increasingly naturalistic conditions, namely the acute neurophysiological response to alcohol [9]-[13]. An analytic method developed to measure drug effects by combining EEG and task performance measures into a single score [14] was adapted for use here on EEG measures by themselves. Different groups of subjects were used in the three studies to help assure that the findings were not idiosyncratic to the small groups of individuals studied.

## Methods

### Participants

#### Ethics

All studies were conducted according to the principles expressed in the Declaration of Helsinki. All studies were reviewed and approved by The SAM Human Subject’s Research Institutional Review Board (Department of Health and Human Services Registration Number DHHS IRB00000816), and all participation was fully informed and voluntary with written consent.

#### Study 1

Participants were 15 healthy adults (21–32 years, mean age 26, 8 women). Health status of subjects was assessed by questionnaire. Participants reported having no current or past neurological or psychiatric disorders, were non-smokers and were light to moderate users of alcohol (1 to 12 drinks per week). Participants with recent or habitual use of illicit drugs or medications that may interact with alcohol were excluded.

#### Study 2

Participants were 15 healthy adults (21–58 years, mean age 31, 8 women), none of whom participated in Study 1. Participants were licensed drivers, light to moderate users of alcohol (1 to 10 drinks per week), and had the same health status and inclusion/exclusion criteria as in Study 1. Prescription and non-prescription medications ingested in the prior 48 hours were recorded and several participants reported having taken multivitamins, aspirin or ibuprofen.

#### Study 3

Ten scientists, engineers and research associates in our laboratory (5 women) participated in the study. None had participated in Studies 1 or 2. Each participant was a test subject and also helped with data collection. They were light to moderate users of alcohol (1 to 12 drinks per week), ranged in age from 25 to 63 (mean 39) years, and had the same health status and inclusion/exclusion criteria as in Study 1.

### Test Administration

Breath alcohol content (BAC) was measured with a breathalyzer ∼30 minutes post-drinking in all the studies.

#### Study 1

Participants performed a 2-back spatial working memory (WM) task in a block of 50 trials, lasting approximately four minutes, during which they had to decide whether the position of the current stimulus was in the same (match) or different (no-match) location as that of the stimulus presented two trials previously [15]–[19]. In this task, a stimulus (letter of the alphabet) was displayed for 200 ms in one of 12 positions on each trial, every 4.5 s. Participants responded “match” or “no-match” on each trial with the left and right mouse buttons, respectively. Participants also performed an easier version of the WM task in which they had to decide whether the position of the current stimulus was the same as that of the first stimulus in the block of 50 trials. Stimulus and response parameters were the same as in the 2-back task. Participants also performed a zazen control task for 1.5 minutes in which they stared at a foveally presented dot and focused their attention on their ordinary inhalations and exhalations. Data from the WM and control tasks were combined to derive a multivariate metric for assessing alcohol effects during laboratory cognitive testing conditions.

#### Study 2

Participants in this study operated an automobile driving simulator (STISIM Drive; Systems Technology, Inc., Hawthorne, CA) for ∼20 minutes. The participant watched a first-person, out-of-the-windshield view on a 20-inch LCD monitor placed at eye-level ∼2 feet from the eyes and controlled the car using a steering wheel and pedal set (Microsoft SideWinder) with the position adjusted to suit each participant. For each test, participants performed one of 30 different scenarios, with the difficulty and number and position of unpredictable events equated across scenarios. Each scenario could be performed in ∼16–20 minutes and presented a variety of different conditions, ranging from long stretches of highway driving with few other cars to urban driving conditions with heavy traffic, stoplights, pedestrians, and other hazards. The top speed of the simulator was 80 mph and participants were instructed to reach the endpoint of the scenario as quickly as possible without causing accidents and following the rules of the road.

#### Study 3

**Figure 1:**
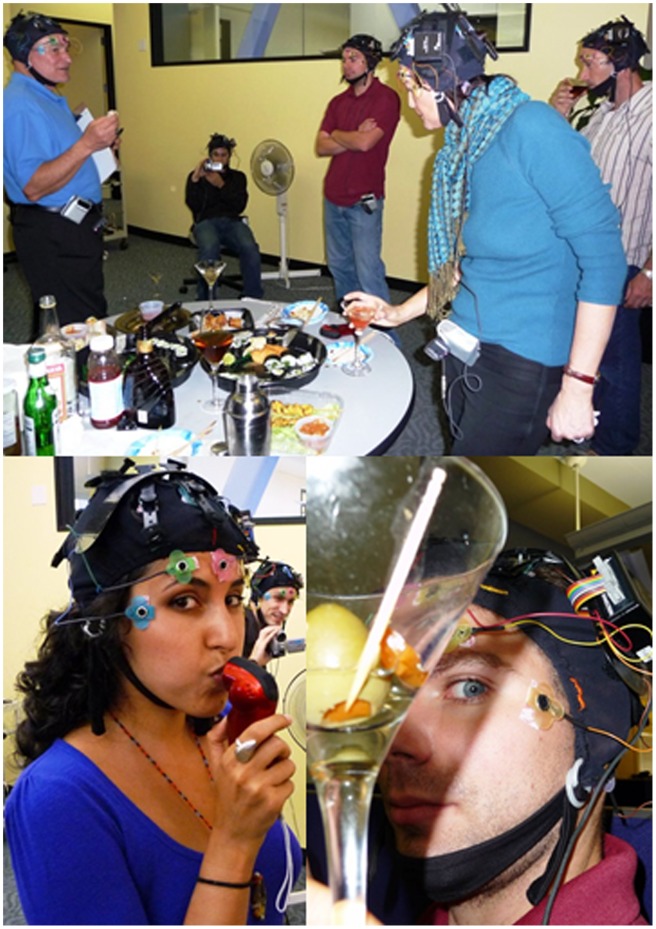
EEGs of ten ambulatory participants simultaneously measured during a cocktail party. EEGs were recorded and transmitted to PCs while the partiers chatted, ate sushi and hors d'oeuvres and drank vodka martinis or vodka and cranberry cocktails according to their personal inclinations. Participants also measured breath alcohol contents, took photos and checked the data collection (lower photos). The six participants shown in photos have given written informed consent, as outlined in the PLoS consent form, to publication of their photograph.

The cocktail party was unscripted, other than withholding drinking for the first 10 minutes to record a pre-drinking baseline. The partiers intermingled, chatted, ate sushi and hors d'oeuvres and drank vodka martinis or vodka and cranberry cocktails according to their personal inclinations. They also measured BACs, took photos and checked up on the automated data collection (Figure 1).

### EEG Recording

In Study 1, EEGs were recorded from 40 electrodes, including the sites measured in Studies 2 and 3, using a stretchable cap with gel-filled electrodes. Signals were sampled at 256 Hz and band-pass filtered from 0.01 to 100 Hz. Prior to the analysis, sites were selected and the data was digitally filtered and down-sampled to match the data recorded in Studies 2 and 3. In Studies 2 and 3, EEG was continuously recorded with a stretchable nylon cap with disposable solid-hydrogel electrodes placed over bilateral and midline dorsolateral prefrontal locations (F3, F4, Fz), midline sensorimotor cortex (Cz), lateral superior parietal cortex (P3, P4), and midline parieto-occipital cortex (POz), referenced to digitally linked mastoids [20]-[21]. These locations were selected, based on cognitive EEG studies with 40 or 100 electrodes [16], for their sensitivity to variations in WM task difficulty. Vertical and horizontal eye movements were monitored with electrodes placed above and lateral to each eye. Signals were amplified, digitized at 128 Hz, band-pass filtered from 0.1 to 35 Hz and transmitted to a PC by an electronic module on the cap. The cap and its electronics were made in our lab and are not commercially available.

### Procedures

#### Studies 1 and 2

Each participant completed alcohol and placebo test sessions, occurring at least one week apart and conducted according to a double blind, randomized, counter-balanced design. In each session, participants consumed a fruit juice drink containing either 0.88 g/kg of 95% ethanol calculated to produce a peak BAC approaching 0.08 grams per 210 liters of breath (the legal limit for operating a motor vehicle in California), or with 5 ml of alcohol floated on top to mimic the smell and taste of the treatment drink. Participants were tested during a testing interval that began 30 minutes after drinking, corresponding with the ∼30–90 minute time range of peak BAC [22].

#### Study 3

Teams placed headsets simultaneously on groups of participants; total set up time was about a half hour. Data from each EEG headset was transmitted during the party via Bluetooth protocol to its own dedicated notebook computer. The data were time synchronized across computers by a start signal sent via local Ethernet from one computer to all other recording computers.

### Data Analysis

#### EEG spectral analysis

For Studies 1 and 2, EEG data during task performance was analyzed from the testing interval that began 30 minutes after drinking, amounting to ∼10 min in Study 1 and ∼20 min in study 2. For Study 3, ∼3 minutes before drinking and ∼3 minutes beginning 30 minutes after drinking was analyzed. Automatic detection and removal of artifacts due to eye movements and blinks, scalp muscle activity, head and body movements, and bad electrode contacts [23]-[24] was followed by visual inspection of all decontaminated and raw data. Power spectral estimates were computed from Fast Fourier Transforms of the decontaminated, Hanning-windowed EEG data of each electrode of each participant by averaging the 50% overlapped 2-second periodograms over a task(s) in Studies 1 and 2, or over the two ∼3 minute intervals in Study 3. The individual frequency component powers were then averaged into 3 standard bands, theta (4–7 Hz), alpha (8–13 Hz) and beta (13–18 Hz) determined in prior studies to be sensitive to alcohol's effect on the EEG [9]-[12], [18], [25]. The 3 banded powers and their standard deviations constituted a total set of 6 variables for each of the 7 electrode sites. For each study, the power and standard deviation values were transformed to z-scores within subjects across alcohol and placebo or pre-drinking conditions to reduce scale differences between the variables and between subjects [26], [27]. The values of the 6 variables at the 7 electrode sites were then averaged over all sites to produce 6 final candidate variables characterizing alcohol’s effect over the entire cerebral cortex [14]. Differences in the variables between alcohol and placebo/pre-drinking conditions were assessed for each study by ANOVAs with condition and variable as within-subjects factors. Degrees of freedom in the ANOVAs were adjusted when appropriate using the Greenhouse-Geisser technique to correct for violations of the sphericity assumption.

#### Multivariate divergence analysis of the EEG effects of alcohol

The subset of EEG variables from the 6 candidates which best discriminated alcohol from placebo conditions was identified in an exploratory analysis of the data from Study 1. The resulting combination of variables and their weighting in an equation was then tested on data from Studies 2 and 3 to assess generalization to the more complex simulated driving task and to the real world situation of a group of people interacting socially. Application of divergence analysis to produce such an equation is described in detail in Gevins et al. [14] and is briefly summarized here.

#### Study 1 exploratory analysis

For each participant, the values of the 6 candidate variables during cognitive testing for both alcohol and placebo sessions were submitted to a multivariate divergence analysis, a simple type of discriminant analysis that searched for the subset of variables producing the largest divergence between alcohol and placebo data [28], [29]. The divergence analysis considered all possible subsets of up to 3 of the 6 variables and chose the subset that, considered together as a group, best recognized alcohol. This resulted in an equation which produced a score that could be used to classify data samples from an individual as being either from alcohol or placebo/pre-drinking conditions.

#### Study 2

Data during driving simulator operation from alcohol and placebo sessions were tested with the equation computed from Study 1. For each participant, for alcohol and placebo conditions, the values of each of the variables chosen in Study 1 were weighted by multiplying it by the percentage of the total divergence contributed by that variable in Study 1 and dividing by its variance in Study 1. The resulting values were then summed with a constant to form an EEG score quantifying the neurophysiological effect of alcohol and a score for placebo. EEG scores from each study were scaled such that the mean of scores from Study 1 were −0.5 in the alcohol condition and +0.5 in the placebo condition. A relatively negative score in the alcohol condition indicated that the individual was affected by alcohol in a similar manner to that of participants in Study 1. A relatively positive score in the alcohol condition indicated that the individual had variable values more typical of the placebo condition in Study 1. Corresponding interpretations would apply for relatively positive and negative scores in the placebo condition.

#### Study 3

The equation from Study 1 was similarly applied to the pre- and post-drinking data from each cocktail party participant to produce EEG scores for that individual.

Thus, for each study, data from each participant were combined into EEG scores in the alcohol and placebo/pre-drinking conditions, using the up to 3 variables, weights, and variances calculated in the divergence analysis in Study 1. Then for each study, participants’ post-drinking and placebo/pre-drinking scores were compared using a receiver-operator characteristic (ROC) analysis to assess the significance of recognition of alcohol [30]. Area under the ROC curve (AUC), sensitivity (correctly identifying the alcohol condition), and specificity (correctly identifying the placebo/pre-drinking condition) values were computed. The significance of the AUC was then determined by transforming the AUC into a z-score with respect to the null hypothesis of AUC = 0.5, and referencing the z-score to the normal distribution, i.e. z score = AUC minus 0.5 divided by the standard error for AUC = 0.5. Pearson correlations were computed to assess the correlation of the EEG divergence scores to BAC values.

## Results

### Breath Alcohol Content

The average BAC 30 minutes after drinking was.07 (sd.02) in Study 1,.07 (sd.01) in Study 2 and.07 (.02) in Study 3 (values from later in the session were conservatively interpolated for several subjects due to missing/invalid readings at that time point).

### Task Performance

In Study 1, accuracy was not affected by alcohol in either WM task (easy WM t(1,14) = 0, p>.05; difficult WM t(1,14) = −1.37, p>.05), nor was reaction time (easy WM t(1,14) = −.34, p>.05; difficult WM t(1,14) = .40, p>.05). In Study 2, driving simulator performance was not affected by alcohol (lane position deviations t(1,14) = .43, p>.05; driving off road incidents t(1,14) = 2.1, p>.05; collisions t(1,14) = 2.0, p>.05). The partiers in Study 3 intermingled amiably, consumed cocktails and sushi to their content, chatted, made jokes, and laughed a lot. It was a good party.

### EEG

In Study 1, ∼96% of the cognitive task data was valid and ∼4% was lost to unrecoverable artifact, resulting in ∼9 minutes of data for analysis from each participant for each of the placebo and alcohol sessions. In Study 2, ∼96% of the driving simulator data was valid and ∼4% was lost, resulting in ∼18 minutes of driving simulator task data for analysis from each participant for each of the placebo and alcohol sessions. In Study 3, ∼60% of the data was valid and ∼40% was lost, resulting in 2.75 to 3.35 minutes of valid pre- and post-drinking data for each participant.

**Figure 2:**
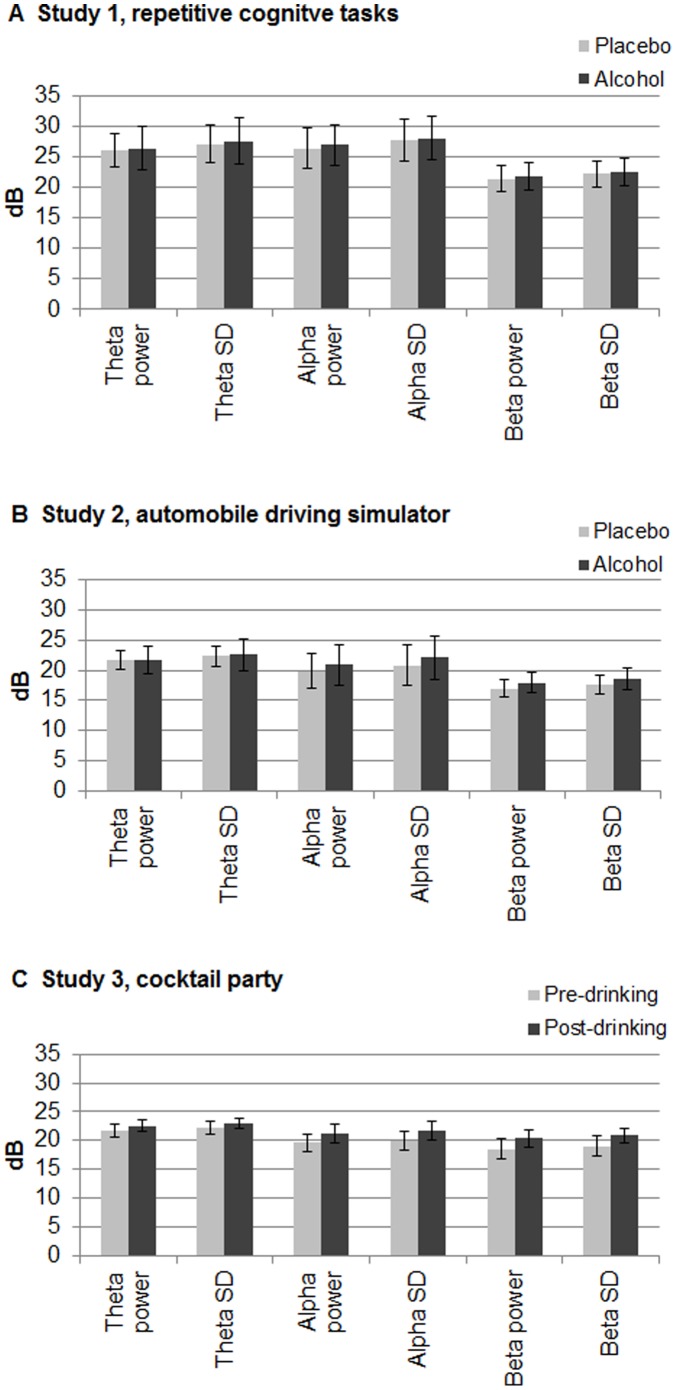
Mean power values for the six candidate EEG variables . Means over participants of EEG power and within-participant standard deviation of power in theta, alpha and beta bands for alcohol (dark bars) and placebo or pre-ingestion (light bars) conditions for the three studies. These variables were not affected by alcohol in (A) Study 1 (p>.05), but were in (B) Study 2 (p<.01) and (C) Study 3 (p<.01); the four alpha and beta variables increased with alcohol in Study 3 (p’s<.05). In an exploratory analysis of Study 1, the divergence analysis selected beta and theta band power from the group of six, and weighted and combined them in a simple equation that recognized alcohol’s EEG effect. That equation was then applied to the different groups of participants in Studies 2 and 3. Error bars are standard deviation.

Figure 2 shows the EEG power and standard deviations in theta, alpha and beta bands of the alcohol and placebo/pre-drinking conditions for the three studies. There was no significant difference between the alcohol and placebo conditions in Study 1 (F(1,14) = 1.92, p>.05), but the variables were significantly different in Studies 2 and 3 (F(1,14) = 10.22, p<.01 and F(1,9) = 18.20 p<.01, respectively). There was a significant condition × variable interaction for Study 2 (F(1,20) = 4.82, p<.05), but post-hoc comparisons did not reveal significant differences between alcohol and placebo/pre-drinking for any of the variables. The condition x variable interaction was also significant for Study 3 (F(2,15) = 5.04, p<.05), where power was greater in the alcohol than in the placebo/pre-ingestion condition for the alpha and beta power and SD variables (F(1,19) = 5.39, p<.05; F(1,19) = 5.85, p<.05; F(1,19) = 6.68, p<.05; and F(1,19) = 6.92, p<.05 respectively).

In the Study 1 exploratory analysis, the highest divergence between alcohol and placebo was produced by a combination of 2 measures from the candidate group of 6: increased beta band power and decreased theta band power in the alcohol condition. The equation was as follows:
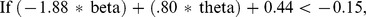
then the segment was classified as being from the alcohol data sample.

This resulted in an AUC of 0.73 (z (29) = 2.14, p<.05), with 67% sensitivity and 87% specificity in distinguishing alcohol from placebo (Table 1, left column).

**Table 1:** Neurophysiological effects of alcohol.

	Study 1: Cognitive Tasks	Study 2: Simulated Driving	Study 3: Cocktail Party
Placebo/Pre-drinking EEG score	0.5 (.72)	.44 (.85)	1.08 (1.19)
Post-drinking EEG score	−0.5 (1.96)	−2.16 (1.38)	−.78 (.56)
AUC (95% CI)	0.73 (.53–.93)*	0.95 (.87–1)***	0.87 (.68–1) **
Sensitivity (%)	67	93	80
Specificity	87	73	80

Rows show mean (standard deviation) of EEG scores for placebo/pre-drinking and post-drinking conditions, the Area Under the Curve (AUC) (95% confidence interval) for recognizing post-drinking from placebo or pre-drinking, the sensitivity of recognizing alcohol and the specificity of recognizing the placebo or pre-drinking conditions.

* p<.05,

** p<.01,

*** p<.0001.

Applying the equation to the Study 2 data, sensitivity in recognizing the post-drinking alcohol data was 93% and specificity in recognizing the placebo data was 73% (AUC 0.95, (z (29) = 4.21, p<.0001) (Table 1, middle column). Applying the equation to the cocktail party data, sensitivity in recognizing the post-drinking alcohol data was 80% and specificity in recognizing the pre-drinking data was 80% (AUC 0.87; z (19) = 2.80, p<.01) (Table 1, right column).

#### Individual differences in EEG alcohol responses

**Figure 3:**
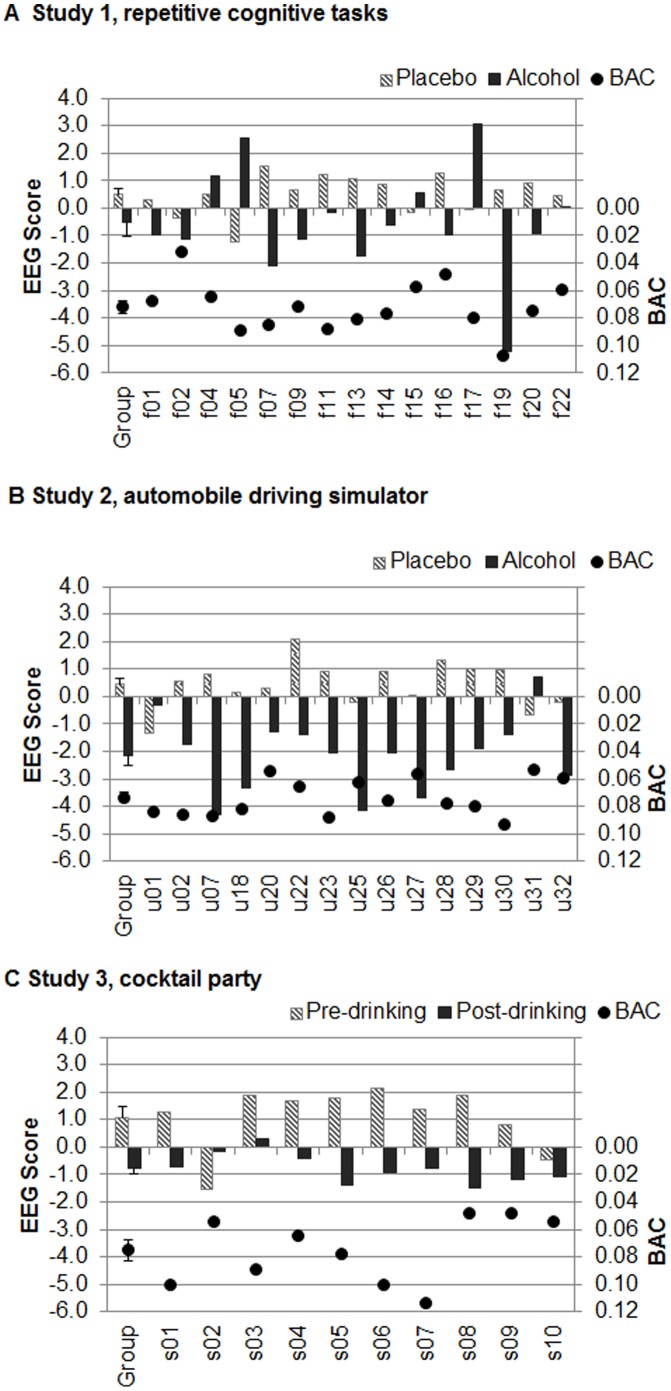
Individual responses for the 40 participants in the three studies. (A) Study 1, repetitive cognitive tasks, (B) Study 2, automobile driving simulator, and (C) Study 3, cocktail party. Left scale: EEG scores after drinking alcohol (dark solid bars) and placebo/pre-drinking (light stippled bars) conditions. Right scale (inverted): Breath Alcohol Contents (BACs – dots). The EEG scores were computed by applying the equation from Study 1 to each of the 40 participants. The leftmost two bars and dots in each panel show the average scores (with standard error bars) for the respective group. The EEG scores were significantly related to BAC in all 3 studies. Although the alcohol condition had lower EEG scores than the placebo/pre-drinking condition for the groups as a whole, there were considerable individual differences.

Each participant’s EEG alcohol scores in the three studies are shown in Figure 3. For Study 1 during cognitive tasks, 11 of the 15 participants showed the same pattern as the group with lower scores for alcohol than placebo, while 4 had the reverse response. EEG scores were negatively correlated with BAC (*r = −.38*, p<.05). During simulated driving in Study 2, 13 of the 15 had the same pattern as the group, again with a negative correlation between EEG scores and BAC (*r = −.75*, p<.001). For Study 3, 9 of the 10 partiers had the same pattern as the group, with a negative correlation between EEG scores and BAC (*r = −.64*, p<.01).

## Discussion

Initial progress is reported towards measuring the effects of a psychoactive substance on the brain function of an entire group of ambulatory people measured simultaneously during a real world activity. An incremental approach was used in which an equation was generated to assess alcohol effects on the EEG during cognitive testing in a double-blind, placebo-controlled experiment, 30–50 minutes after drinking when breath alcohol concentrations were just under the California legal limit for driving. The resulting equation distinguished alcohol from placebo with a simple weighted combination of beta band and theta band power measures. The equation was then applied, with good results, to a second double-blind, placebo-controlled study of alcohol effects on a different group of participants performing the more complex task of operating an automobile driving simulator. Finally, the same equation readily recognized the effect of alcohol in the third group who were having a good time at a cocktail party.

Alcohol did not affect working memory task performance in Study 1 or automobile driving simulator performance in Study 2. Values for theta, alpha and beta band power and standard deviation variables were not affected by alcohol in Study 1, but were affected in Studies 2 (p<.01) and 3 (p<.01). These differences in EEG ANOVA significances between the three studies may merely reflect chance variations in the small subject populations. In Study 3, post-hoc tests showed significantly greater power and standard deviations for the alpha and beta variables, consistent with prior studies [9]–[13], [18], [25], [31]. Thus, the equation generated on EEG data with a weak alcohol effect performed well when applied in the two independent studies with the same BAC level. This suggests that the equation indeed quantified a salient aspect of the neurophysiological effect of alcohol.

With few exceptions [14], prior EEG research on alcohol effects during cognitive performance has not focused on individual variations in response to alcohol. In the current study, individual differences were assessed by applying the same equation to the data of each of the participants. There was a significant negative correlation between BAC measures and the individual EEG scores in all three studies. While the EEG scores for most individuals were relatively more negative after drinking and relatively more positive in the placebo condition or before drinking, there were individual variations, more so in Study 1. While some of these individual differences are interesting (e.g. the two subjects in Study 2, u01 and u31, with the best driving performance had driving EEG scores that were markedly less affected by alcohol than the group), the small number of subjects precludes further discussion of such findings until further research is undertaken.

A major concern in measuring brain function from active ambulatory people is loss of data because of unrecoverable contamination due to artifacts. Automated methods for artifact detection and decontamination have improved over the years [23], [24], [32]–[36] and are helpful in reducing human labor, improving consistency and recovering some of the data that would otherwise have to be discarded. However, such methods are still far from perfect and it is still essential that all data be subjected to labor-intensive expert manual review. In the current studies, data attrition increased markedly when recording under real world conditions. In Studies 1 and 2 in which participants were seated, stared straight ahead at a video monitor, made constrained limb movements and did not talk, ∼4% of the data to be analyzed was lost. By contrast, in Study 3, ∼40% of the data to be analyzed could not be recovered due to artifacts generated by chewing, swallowing, talking and moving around during the cocktail party. Yet, even so, it is encouraging that the majority of the data was valid under such conditions. Future improvements in automated artifact decontamination methods will hopefully produce higher rates of valid data.

The results reported here are merely a pilot study that helps to establish the feasibility of validly measuring the neurophysiological effects of a psychoactive substance in real world conditions from groups of people simultaneously. We do not wish to attach too much significance to the specific findings. Given the small number of participants, regional cortical effects of alcohol were not examined because the large number of variables produced by including data from each electrode site in the divergence analysis would have produced an unreliable equation that over fitted the data [37]. Rather, a global effect on the cerebral cortex was obtained by averaging the data from the individual electrodes. Application of principal components analysis [38], statistical pattern recognition methods [39], [40], global field power [41], [42], independent components analysis [43], etc. might be more effective. Future studies with larger subject populations might fruitfully explore differential effects on frontal, parietal and other cortical areas, for instance using high resolution EEG methods with many electrodes, spatial deblurring and MRI co-registration [44]. Amongst the many types of analysis available, the simplest form of spectral analysis into traditional frequency bands was used to parameterize the EEG. The beta band and theta band EEG power variables chosen by the divergence analysis may not be the best measures of alcohol’s effect during naturalistic situations. They are merely variables that were most effective in combination when characterizing alcohol effects 30 minutes after drinking during performance of repetitive cognitive tasks in one sample of healthy adults. Other analyses such as, time-frequency analysis, independent component analysis, autoregressive models of various sorts, nonlinear dynamical models, etc. might be more effective.

Modest though they are, these initial results promisingly suggest that future studies may be able to apply brain function measures of drug effects derived in rigorously controlled laboratory neurocognitive research to groups of people interacting in natural social situations. By doing so, we hope that the effects of alcohol and other drugs on particular aspects of social interaction will be elucidated with a focus on the fine grain structure of interactions between individuals and relationships to varying doses and pharmacodynamics. In addition to advancing basic knowledge, such future research may provide useful information for improving pharmacological treatments of a variety of psychiatric disorders.
